# Aerobic exercise therapy in severe mental disorders: from methods to underlying mechanisms

**DOI:** 10.1007/s00406-025-01991-4

**Published:** 2025-03-14

**Authors:** Andrea Schmitt, Lukas Roell, Isabel Maurus, Peter Falkai

**Affiliations:** 1 Department of Psychiatry and Psychotherapy, LMU University Hospital, Ludwig-Maximilians- University Munich, Nussbaumstraße 7, 80336 Munich, Germany; 2 Max Planck Institute of Psychiatry, Kraepelinstrasse 2-10, 80804 Munich, Germany; 3 Laboratory of Neuroscience (LIM27), Institute of Psychiatry, University of Sao Paulo, Rua Dr. Ovídio Pires de Campos, 785, São Paulo, 05403-903 SP Brazil

Schizophrenia, bipolar disorder (BD) and major depression (MDD) are summarized as severe mental illnesses (SMIs) and characterized by a variety of different symptoms such as anhedonia, delusions and hallucinations. From a transdiagnostic perspective, especially cognitive deficits, impaired social and occupational functioning, and substantial decline of somatic health, e.g. metabolic syndrome play a crucial role across all of these disorders. Hoertel et al. [1] compared older adults with schizophrenia to BD and MDD with regard to 5-year mortality and its causes. Schizophrenia compared to MDD or BD was significantly associated with increased all-cause mortality and cardiovascular mortality. These associations were significantly reduced among patients taking antidepressants. Current treatment options, which comprise antidepressant and antipsychotic medication, psychotherapy, cognitive remediation, and non-invasive brain stimulation, differ with regard to their therapeutic windows and the underlying treatment goals. Although these interventions have been proven to be effective, there is still great potential to improve long-term disease outcomes in people with SMI. For instance, a current meta-analysis in schizophrenia shows that only 24.2% of the patients show recovery, and only 35.5% had a good or better outcome [2]. Especially cognitive deficits and associated deteriorations in social and occupational functioning often persist over a long period of time. Consequently, there is an urgent need to further improve current treatment options in SMIs.

In recent years, lifestyle interventions, encompassing treatments such as physical exercise therapy, have been shown to represent efficient add-on therapies in SMI. Beyond further improvements in residual core symptoms, these treatments yield the potential to specifically address cognitive deficits, social and occupational functioning, and somatic health.

In a randomized controlled trial in patients with schizophrenia spectrum disorder, subjective physical fitness parameters were assessed before and after exercise and control sessions. Weekly physical activity in patients with schizophrenia spectrum disorder was lower than in healthy controls and attributed to reduced engagement in sport activities. Compared to healthy controls, the relationship between subjective and objective physical fitness parameters in patients with schizophrenia spectrum disorder was missing and may represent a barrier for stronger engagement in physical activity. However, in the patient group, during exercise sessions subjective physical fitness ratings increased to a stronger extent than in healthy controls [3]. This indicates that patients may benefit from structured and supervised exercise training. In another randomized study, 31 schizophrenia patients were assigned to either a controlled endurance training consisting of 20–30 min training 3 times per week for a period of about 2 months or 90 min occupational therapy 2–3 times per week. Significant improvements in cognitive functions and psychopathology could be shown in both groups. However, for some memory functions (short-term verbal memory, working memory, and learning performance), there was a significant advantage for the aerobic endurance training group [4]. This is in line with a previous multicenter study showing improvement in cognitive performance and an increased volume of hippocampal subfields after endurance training in people with schizophrenia [5, 6]. A randomized controlled trial aimed to study a physical aerobic and muscle-strengthening exercise intervention compared to bright light therapy (home-based exposure of white light provided by a 10,000 lx light box) guided via a mobile health app in young people with attention-deficit/hyperactivity disorder. Instruction, monitoring and feedback were realized with a smartphone equipped with the m-health app system. In contrast to replicated effects of aerobic exercise using in person supervised training, the application of the app revealed no effects on depressive symptoms. During the intervention period of 10 weeks, the wearing time of the wrist-worn mobile sensor to record online physical activity and light exposure dropped to 30.1% in the bright light therapy group and to 41.4% in the exercise intervention group, and intervention adherence (≥ 80% completed exercise or bright light therapy sessions) was low [7]. This study shows that the single use of an app-based intervention may reach its limits in this group of patients.

Despite increasing evidence for health-promoting effects of aerobic exercise training in people with SMI, the underlying mechanisms still have to be elucidated. Playing an important role in energy supply, mitochondria are a key candidate of mechanistic understanding. Shi et al. [8] conducted mitochondria-wide association studies (MiWAS) to assess the association of mitochondrial Single Nucleotide Polymorphisms SNP with each aspect of subjective well-being using data from the UK Biobank. Additionally, an interaction analysis of mitochondrial DNA (mtDNA) mutation and physical activity was performed to evaluate their joint effect on the subjective well-being status. MiWAS analysis identified 45 mitochondrial SNPs associated with 9 phenotypes of subjective well-being. They also identified 10 significant mtDNA-physical activity interaction sets for subjective well-being. Interestingly, in mtDNA-physical activity interactions they described 7 mtDNA affecting psychiatric disorders.

In schizophrenia, a decreased number of oligodendrocytes and evidence for disturbed myelination has been described in several brain regions, such as the CA4 subregion of the hippocampus and the prefrontal cortex [9]. In this issue, Kolomeets and Uranova [10] describe a significant decrease of the number of satellite oligodendrocytes per neuron in the head of the caudate nucleus in individuals with schizophrenia as compared to healthy controls, extending previous findings of reduced satellite oligodendrocytes in the prefrontal and parietal cortex. The association of reduced oligodendrocytes with cognitive deficits in schizophrenia patients led to the hypothesis that the decreased number of oligodendrocytes is related to a failure of maturation and indicates a disturbed regenerative recovery process in distinct brain regions [9]. Since the hippocampal CA4 subfield volume increased during endurance training [6], aerobic exercise may foster such a regenerative process. In addition to synaptic mechanisms, future studies should also focus on the role of mitochondria, oligodendrocytes and the differentiation of their precursor cells.
